# Elucidating the Influence of Sodium Fluoride on the Structural and Photoelectrochemical Properties of Antimony Sulfide Thin Films

**DOI:** 10.1021/acsomega.5c09509

**Published:** 2026-01-16

**Authors:** Kavya Dodderi Manjappa, Akshay Kumar Sonwane, Sajan Daniel George, Raviprakash Yeenduguli

**Affiliations:** † Department of Physics, Manipal Institute of Technology, Manipal Academy of Higher Education, Manipal 576104, India; ‡ Department of Metallurgical Engineering & Materials Science (MEMS), 226957 Indian Institute of Technology, Indore 453552, India; § Centre for Applied Nanosciences, Manipal Institute of Applied Physics, 76793 Manipal Academy of Higher Education, Manipal 576104, Karnataka, India

## Abstract

Photoelectrocatalysis has become a sustainable and eco-friendly method for water splitting, enabling the production of hydrogen and oxygen using solar energy. Among various photoabsorber materials, antimony sulfide (Sb_2_S_3_) is regarded as a promising candidate for photoelectrochemical (PEC) water splitting due to its suitable band gap (∼1.7 eV), high optical absorption coefficient, and the earth-abundant nature of its elements. However, its practical application is limited by deep-level defects that can cause charge carrier recombination and reduce PEC efficiency. This work investigates how sodium fluoride (NaF) doping influences the structural, optical, and electrochemical properties of Sb_2_S_3_ thin films. NaF doping modifies the surface morphology and decreases surface roughness. These structural changes are associated with an increase in photocurrent density from 0.48 to 0.52 mA cm^–2^ and a significant rise in carrier concentration from 9.24 × 10^15^ to 3.47 × 10^18^ cm^–3^ under standard illumination. These results demonstrate that NaF plays a key role in controlling charge transport and improving catalytic activity. Overall, the findings highlight how dopant-induced modifications affect the PEC performance of Sb_2_S_3_-based photoelectrodes.

## Graphical abstract

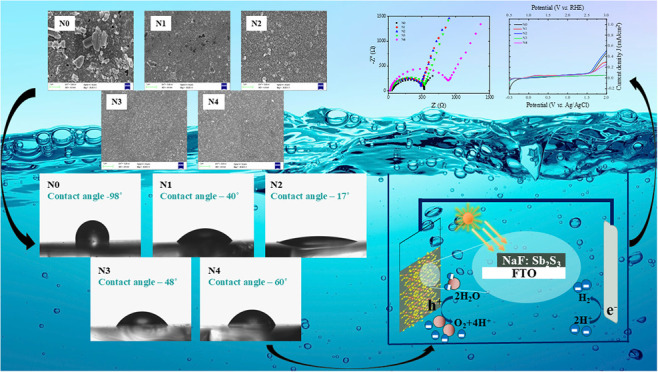

## 1 Introduction

Antimony sulfide (Sb_2_S_3_) has attracted considerable interest in recent years as a promising absorber material for high-efficiency photovoltaic and photoelectrochemical (PEC) devices.^1–3^ It features a direct band gap ranging from 1.6 to 1.8 eV, suitable for visible light absorption and closely aligned with the solar spectrum. This enables Sb_2_S_3_ to function effectively as a top subcell in tandem structures with crystalline silicon (c-Si), potentially reaching a theoretical power conversion efficiency (PCE) of over 40%.^4^ Besides its appropriate band gap, Sb_2_S_3_ offers several advantages, including high optical absorption (greater than 10^5^ cm^–1^ in the visible range), low manufacturing costs, and the use of earth-abundant, nontoxic elements.^5–7^ Under standard AM 1.5G solar illumination, it can attain a theoretical photocurrent density of up to 24.5 mA/cm^2^, corresponding to a solar-to-hydrogen (STH) efficiency of approximately 28%.^8^ These properties make Sb_2_S_3_ an ideal candidate for PEC water splitting applications, either as the main light absorber or as a surface sensitizer.

Despite these advantages, the practical use of Sb_2_S_3_ is limited by several factors, such as rapid photocorrosion, poor charge carrier mobility, a high density of surface defects, and inefficient separation of photogenerated electron–hole pairs. Additionally, pristine Sb_2_S_3_ typically exhibits high electrical resistivity and requires a substantial applied bias to initiate water splitting reactions. These issues cause significant recombination of electron–hole pairs, which reduces the photocatalytic efficiency.^9–11^ Therefore, overcoming these limitations is crucial to use Sb_2_S_3_ in PEC and photocatalytic applications effectively.

To address these challenges, several approaches have been investigated, among which doping is a widely accepted and effective method.^12,13^ By introducing suitable dopant atoms into the crystal lattice of a semiconductor, it is possible to modify its electronic structure, create intermediate energy levels, and facilitate the separation and migration of charge carriers. These photoexcited charges can then reach the surface and participate in redox reactions, improving photocatalytic degradation efficiency and reducing recombination losses.,^14,15^

Recent studies have shown that doping Sb_2_S_3_ with elements such as Bismuth (Bi),^16^ Ruthenium (Ru),^17^ Indium (In),^17^ Silver (Ag),^18,19^ and rare-earth (RE) elements can enhance photocatalytic and PEC performance. For example, Europium (Eu) doping improved crystallinity and suppressed recombination at an optimal doping level of 4 at%, resulting in 98.2% degradation of Rhodamine B (RhB) under visible light.^20^ Similarly, ruthenium (Ru) doping at 6 at% significantly enhanced photocurrent density (3.35 mA/cm^2^) and stability over 5 h of PEC testing, due to improved charge transport and a broader band gap (1.72 eV).^17,21,22^ Building on these findings, doping Sb_2_S_3_ with sodium fluoride (NaF) may offer another effective strategy to improve its photoelectrochemical performance. Sodium (Na) and fluorine (F) atoms are expected to influence the material’s conductivity and band alignment, potentially enhancing charge separation and transport.^23–26^ As Na and F are more abundant and less environmentally challenging than rare earth elements, NaF doping provides a cost-effective and scalable approach.

In the present study, sodium fluoride-doped antimony sulfide (NaF: Sb_2_S_3_) thin films were investigated, for the first time, as bare photoanodes for photoelectrochemical (PEC) water splitting. In contrast to previous reports, where Sb_2_S_3_ was primarily employed as a photocathode or required integration into complex heterostructures, this work demonstrates the direct applicability of NaF-doped Sb_2_S_3_ as a standalone photoanode. Systematic variation of NaF doping in Sb_2_S_3_ thin films leads to the formation of secondary phases, specifically sodium sulfide (Na_2_S) and antimony trifluoride (SbF_3_). Na_2_S helps to passivate surface defects, suppressing electron–hole recombination and promoting more efficient charge separation. At the same time, SbF_3_ contributes to lattice reorganization and modifies the local electronic structure, enhancing crystallinity and charge carrier mobility. The overall effect of NaF doping can be further explained by the combined action of Na^+^ and F^–^ ions within the Sb_2_S_3_ lattice. The smaller ionic radius of Na^+^ compared to Sb^3+^ allows partial substitution or interstitial incorporation, which alters the lattice structure and reduces internal strain. F^–^ ions can replace S^2–^ or occupy surface sites, creating local dipoles and modifying the electronic environment. These dopant-induced changes influence crystal growth, defect density, and carrier concentration, leading to improvements in structural quality, optical absorption, and charge transport. Together, the formation of secondary phases, lattice modifications, and the introduction of shallow donor states by Na^+^ and F^–^ ions result in higher carrier concentration and enhanced photoelectrochemical performance in the NaF-doped Sb_2_S_3_ thin films.

This study provides a detailed analysis of how NaF doping influences the structural, morphological, optical, and electrochemical properties of antimony sulfide (Sb_2_S_3_) films. Additionally, it correlates these modifications with changes in surface wettability and interfacial defects that play critical roles in determining PEC efficiency. The observed enhancements in linear sweep voltammetry (LSV) and overall PEC response are attributed to improved light harvesting, increased charge carrier mobility, and reduced recombination losses induced by NaF doping.

## 2 Experimental Details

Antimony sulfide (Sb_2_S_3_) thin films were fabricated using a thermal evaporation technique. Molybdenum (Mo) boat was used as the source holder, and fluorine-doped tin oxide (FTO)-coated glass substrates served as the deposition surface. Before deposition, the substrates were ultrasonically cleaned in isopropyl alcohol for 10 min and subsequently dried using a nitrogen gas stream. High-purity (Sb_2_S_3_) powder (Thermo Fisher, 99.9%) and the cleaned FTO substrates were placed inside a thermal evaporation chamber. The deposition was conducted at an average base pressure of 5 × 10^6^ mbar, achieved with the aid of a liquid nitrogen trap. The source-to-substrate distance was maintained at 16 cm, and the substrate temperature was held at 150 °C throughout the deposition process.

The deposition rate was maintained at 3.5–4 Å/s by adjusting the filament current to 85 A, resulting in (Sb_2_S_3_) thin films with a final thickness of approximately 450 nm. In parallel, sodium fluoride (NaF) layers with thicknesses ranging from 20 to 80 nm were also deposited under similar conditions. Following deposition, the films were subjected to a sulfurization process using a custom-designed chemical vapor deposition (CVD) system. Sulfurization was carried out at 400 °C for 30 min under a vacuum of 900 mbar. Elemental sulfur (100 mg, Thermo Fisher, 99.9998%) was evaporated in a single-zone furnace under a nitrogen atmosphere. After the sulfurization process, the films were cooled to room temperature under vacuum conditions, following the procedure described in our previous study.^27^ Table 1 provides the details of the deposited films.

**Table 1:** Details of the Sodium Fluoride-Doped Antimony Sulfide Thin Films

sample name	thickness (nm) of sodium fluoride (NaF) ± 5 nm
N0	00
N1	20
N2	40
N3	60
N4	80

## 3 Characterizations

The structural properties of undoped and sodium fluoride-doped antimony sulfide (Sb_2_S_3_) thin films were investigated using grazing incidence X-ray diffraction (GIXRD). Measurements were carried out at room temperature with a Bruker AXS D8 Advance diffractometer, employing Cu Kα radiation over a 2θ range of 20–70°, with a grazing incidence angle of 0.5°. The measurements were performed with a step size of 0.02° and a counting time of 1 s per step, corresponding to a scanning rate of 0.1°/min. Raman spectroscopy was conducted in a backscattering configuration using an Nd: YVO_4_ diode-pumped solid-state laser with an excitation wavelength of 532 nm. Surface morphology was examined using scanning electron microscopy (SEM) (Zeiss EVO MA18) operated at an acceleration voltage of 10 kV. Concurrently, elemental composition was analyzed via energy-dispersive X-ray spectroscopy (EDS) using an Oxford INCA X-act detector. X-ray photoelectron spectroscopy (XPS) measurements were performed with a SPECS (Germany) X-ray photoelectron spectroscope utilizing Al Kα (1486.61 eV) radiation and applying a 13 kV anode voltage. Surface topography was characterized by atomic force microscopy (AFM) in tapping mode using a Flex-Axiom AFM system. The surface wettability of the films were characterized via water contact angle measruements. Photoluminescence (PL) measurements were carried out using a fluorescence spectrometer equipped with an Nd:YVO_4_ diode-pumped solid-state laser, with an excitation wavelength of 260 nm.

The photoelectrochemical (PEC) performance of the sodium fluoride-doped antimony sulfide photoelectrodes was assessed using a CompactStat.h potentiostat (IVIUM) in a standard three-electrode PEC H-cell (Redox.me). The deposited film served as the working electrode, a platinum (Pt) wire was used as the counter electrode, and an Ag/AgCl electrode acted as the reference. A 0.5 ∼ M Na_2_SO_4_ aqueous solution (pH = 7) was used as the electrolyte.

The potential versus the reversible hydrogen electrode (RHE) was calculated using the following equation

$$E_{\mathrm{RHE}}=E_{\mathrm{Ag}/\mathrm{agCl}}+0.197\;V+0.059\times \mathrm{pH}$$

where 0.197 V corresponds to the standard potential of the Ag/AgCl electrode versus the normal hydrogen electrode (NHE) at 28 °C.

A 1-Sun LED solar simulator (Redox.me), providing an illumination intensity of 100 ∼ mW/cm^2^, was used as the light source. Illumination was applied from the back side of the electrode, with an exposed area of 0.5 cm^2^.

Linear sweep voltammetry (LSV) and cyclic voltammetry (CV) measurements were carried out at a scan rate of 50 mV/s. Electrochemical impedance spectroscopy (EIS) was performed in the frequency range of 0.1 Hz to 100 kHz using a sinusoidal voltage amplitude of 0.5 V. The Mott–Schottky measurements were performed under dark conditions using an AC frequency of 1 kHz with a modulation amplitude of 50 mV.

## 4 Results and Discussion

### 4.1 Structural Analysis

Figure 1 shows the grazing incidence X-ray diffraction (GIXRD) patterns of both pure and sodium fluoride (NaF)-doped antimony sulfide (Sb_2_S_3_) thin films. The diffraction peaks of all the samples match the standard patterns (JCPD.NO-42–1393) reported in earlier studies for Sb_2_S_3_.^28^ In the pure film, all the expected peaks for Sb_2_S_3_ are seen. Upon NaF doping, diffraction peaks appeared at 23.7, 26.6, 62.7, and 65.9°, corresponding to the formation of antimony fluoride (SbF_3_).^29^ Another peak at 38.8° could be attributed to either sodium fluoride (NaF)^30^ or sodium sulfide (Na_2_S).^31^ There are no peaks related to antimony oxide (Sb_2_O_3_, JCPDS No. 05–0534),^32^ indicating that no oxidation occurred during the doping process. Additionally, the main peaks of Sb_2_S_3_ do not shift after doping, indicating that the crystal structure remains unchanged. This confirms that NaF has been successfully incorporated into the Sb_2_S_3_ lattice without altering its basic structure.

**Figure 1:**
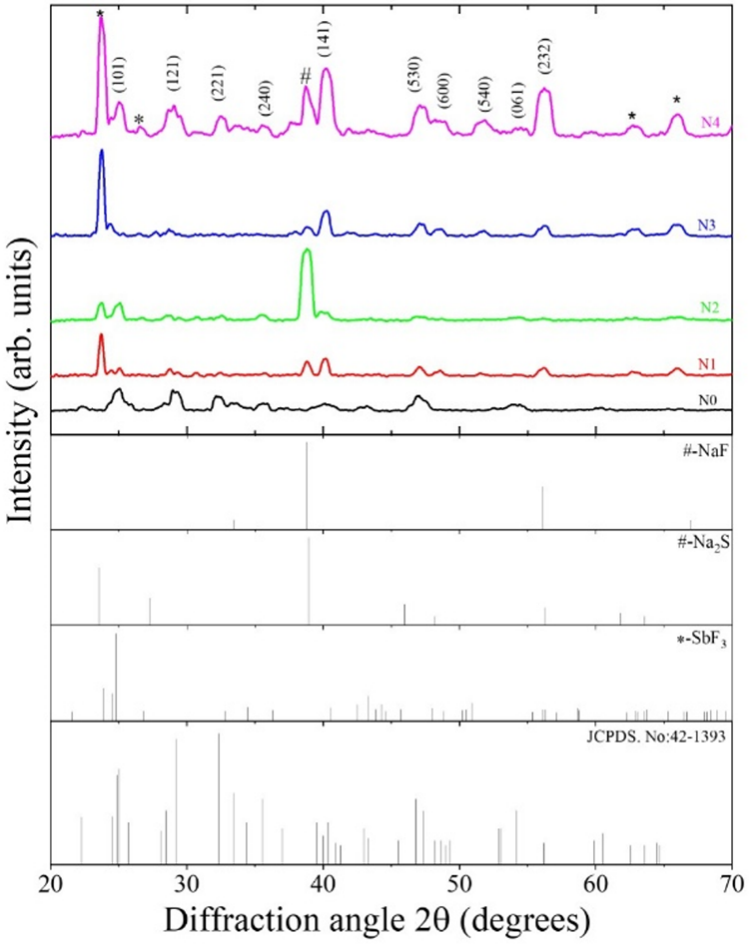
GIXRD patterns of undoped and sodium fluoride-doped antimony sulfide thin films.

Table 2 summarizes the crystallographic parameters obtained from the GIXRD patterns of the Sodium fluoride-doped Sb_2_S_3_ thin films. The sample (N0) exhibits a relatively small crystallite size, indicating limited grain growth. As the doping concentration increases, there is a clear progressive rise in crystallite size, which can be attributed to enhanced grain coalescence and improved crystallinity caused by dopant incorporation.^33^ At the same time, a decrease in microstrain occurs, suggesting that doping helps relieve internal lattice distortions, likely by passivating native defects or encouraging more uniform crystal growth. Furthermore, changes in dislocation density and interplanar spacing (*d*-spacing) are observed, reflecting subtle structural modifications in the Sb_2_S_3_ lattice due to dopant-induced stress relaxation and possible lattice substitution or distortion.^34,35^

**Table 2:** Crystal Parameters of Undoped and Sodium Fluoride-Doped Antimony Sulfide Films

sample	crystallite size (*D* in nm)	microstrain (ε in 10^–3^)	dislocation density (δ in 10^15^/m^2^)	interplanar distance (*d_hkl_*) in Å
N0	16.8	9.6	3.5	3.60
N1	18.3	8.9	3.0	3.64
N2	19.7	8.2	2.5	3.59
N3	22.9	6.5	1.6	3.54
N4	23.2	5.8	1.3	3.55

Figure 2 presents the Raman spectra of undoped and sodium fluoride-doped antimony sulfide (Sb_2_S_3_) thin films. Distinct vibrational modes are observed at 111, 142, 150, 190, 236, 285, 309, and 497 cm^–1^.^27,36,37^ The mode at 497 cm^–1^ corresponds to the fluorine-doped tin oxide (FTO) substrate. The peak at 142 cm^–1^ corresponds to the B_3g_ asymmetric bending vibrations of Sb–S bonds. The mode at 150 cm^–1^ is attributed to A_g_ asymmetric bending vibrations, while the mode at 285 cm^–1^ is due to A_g_ symmetric stretching vibrations. The peak at 309 cm^–1^ is related to B_1g_ asymmetric stretching vibrations of Sb–S.^38,39^

**Figure 2:**
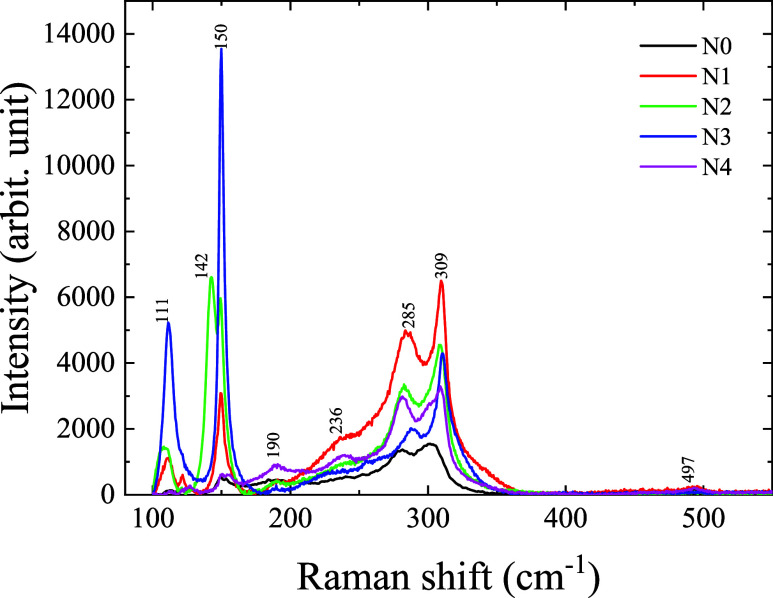
Raman spectra of undoped and sodium fluoride-doped antimony sulfide thin films.

The Raman mode at 111 cm^–1^ is primarily associated with Na–S bending vibrations and may also include contributions from Sb–S symmetric bending vibrations. The mode at 142 cm^–1^, which is observed only in sample N2, can be related to the formation of Na_2_S and Na–F bonds,^40,41^ as well as Sb–S asymmetric bending vibrations. Since these vibrations occur in similar frequency ranges, it is difficult to separate their individual effects. This assignment is further supported by the appearance of a distinct peak at 38.8° in the XRD pattern, which shows higher intensity in sample N2 compared to other samples.

The peak at 190 cm^–1^ corresponds to the symmetric stretching vibrations of Sb–S, suggesting the presence of Sb–F bending vibrations. The mode at 236 cm^–1^ can be attributed to the symmetric stretching of Sb–F bonds.^42^ With increasing NaF doping concentration, noticeable changes in the intensity and sharpness of these Raman peaks are observed. These variations indicate modifications in the bonding environment and crystallinity of the Sb_2_S_3_ lattice, likely due to the incorporation of NaF.

In particular, the modes near 290 and 312 cm^–1^ are correlated with vibrational modes influenced by NaF. The appearance of these modes suggests partial incorporation or surface-level interaction of NaF with the Sb_2_S_3_ matrix. This interaction may lead to alterations in the vibrational behavior and local symmetry of the crystal structure.^43^

### 4.2 Morphological Analysis

Figure 3 shows the scanning electron microscopy (SEM) images of undoped and sodium fluoride (NaF)-doped antimony sulfide (Sb_2_S_3_) thin films, demonstrating the effect of NaF doping on surface morphology. The undoped film (N0) exhibits a relatively porous surface with large and loosely packed grains, indicating uneven nucleation and grain growth during film formation. Upon introducing NaF, noticeable changes in morphology are observed. The sample N2 shows the highest porosity among all compositions, with smaller grain sizes. This increase in porosity at lower doping levels may be attributed to enhanced nucleation and modified growth kinetics facilitated by the presence of fluoride ions. Fluoride ions can alter the surface energy and local chemical environment, promoting faster nucleation and formation of smaller grains with less compact packing.^44^

**Figure 3:**
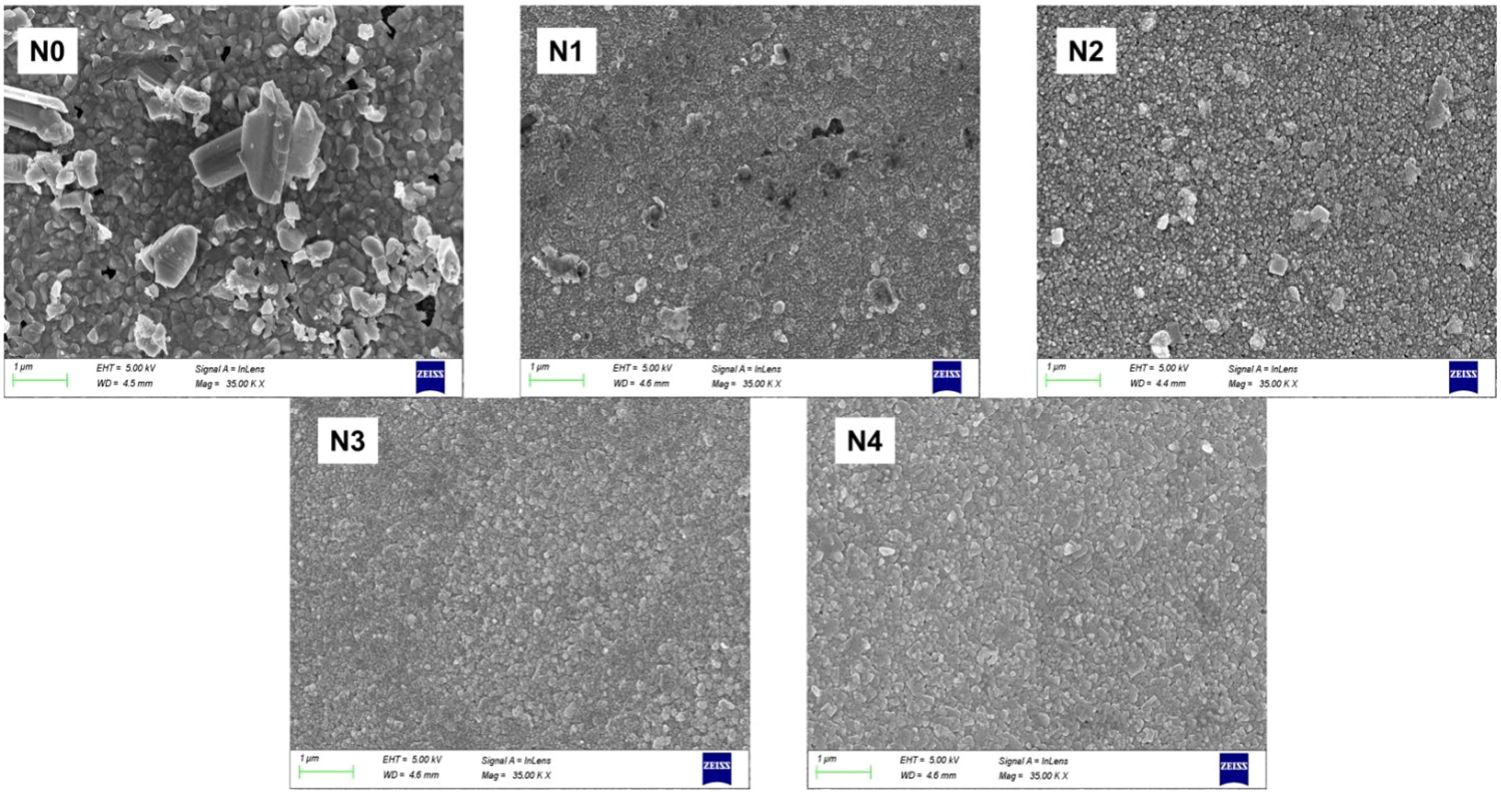
SEM images of undoped and sodium fluoride-doped antimony sulfide thin films.

However, as the NaF concentration increases beyond the N2 composition, the films begin to exhibit a denser morphology with more compact grains and a visible reduction in porosity. This trend suggests that excessive doping leads to saturation of the active nucleation sites and limits grain boundary formation. At higher concentrations, the excess fluoride ions may interfere with uniform grain growth by disturbing the diffusion processes.^45^

Figure 4 displays the atomic force microscopy (AFM) images of sodium fluoride (NaF)-doped antimony sulfide (Sb_2_S_3_) thin films, showing how surface morphology changes with different NaF doping levels. The surface roughness was measured quantitatively using root means square roughness (*R*_q_) and average roughness (*R*_a_) values, as listed in Table 3. These roughness patterns closely match the morphological features seen in the SEM images (Figure 3).

**Figure 4:**
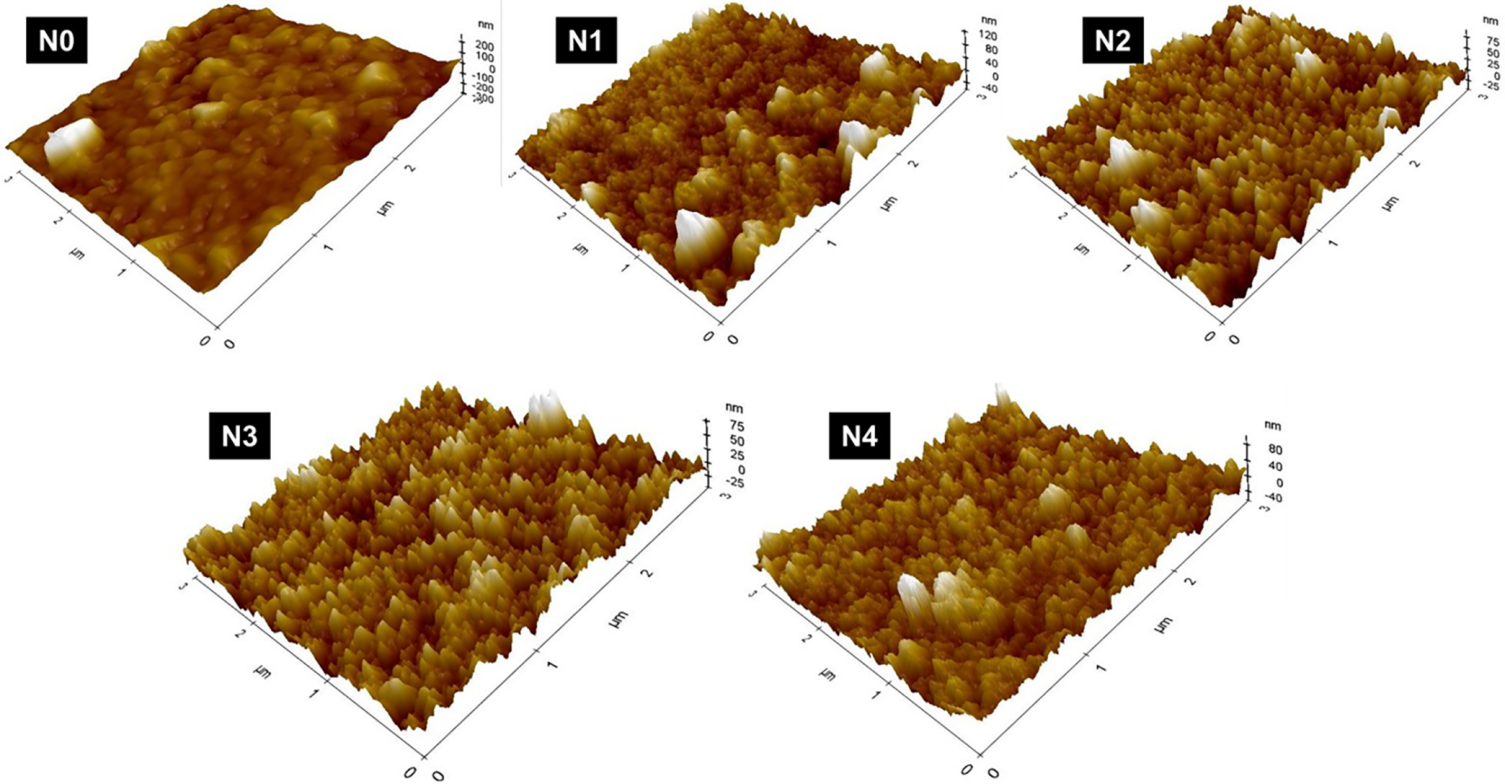
AFM images of undoped and sodium fluoride-doped antimony sulfide films.

**Table 3:** Surface Roughness Parameters of Undoped and Sodium Fluoride-Doped Antimony Sulfide Films

sample name	*R*_q_ (nm)	*R*_a_ (nm)
N0	11.2	14.1
N1	17.5	20.6
N2	24.9	20.8
N3	11.5	14.9
N4	8.3	12.5

The undoped film (N0) shows a smooth surface with low roughness, indicating uniform grain growth and few surface irregularities. As the NaF concentration increases, the surface roughness also rises, peaking at sample N2. This increase in roughness is linked to higher porosity and a greater grain boundary density observed in SEM analysis. The presence of fluoride ions at moderate doping levels likely promotes better nucleation and uneven grain development, resulting in a rougher and more textured surface.^46^

Beyond the N2 composition, a noticeable decrease in surface roughness is seen. This reduction aligns with the SEM findings, where higher doping levels lead to more compact grain structures and reduced porosity. The excess dopant at higher concentrations may inhibit irregular grain growth and encourage densification of the film surface, resulting in a smoother topography with fewer surface features.^47^

### 4.3 Wettability Test

The wettability of undoped and sodium fluoride (NaF)-doped antimony sulfide (Sb_2_S_3_) thin films was evaluated using contact angle measurements, as shown in Figure 5. This method provides insight into the hydrophilic or hydrophobic nature of the film surfaces. A contact angle below 90° indicates a hydrophilic surface, while a value above 90° indicates a hydrophobic surface. Enhanced hydrophilicity is beneficial in photoelectrochemical applications, as it improves the interaction between the electrode surface and the electrolyte, which can facilitate charge transfer and reduce interfacial resistance.^48^ As the NaF doping concentration increases, a gradual decrease in contact angle is observed, indicating an increase in surface hydrophilicity. Among all compositions, the N2 sample exhibits the lowest contact angle of 17°, suggesting the highest degree of wettability. This behavior can be attributed to the influence of NaF doping in the N2 sample, which may lead to the presence of Na_2_S or NaF phases as confirmed by XRD and Raman analyses. The observed trend is consistent with the changes in surface morphology and roughness revealed by SEM and AFM analyses (Figures 3 and 4), respectively. At moderate doping levels, particularly in the N2 sample, the increase in surface roughness and porosity enhances the effective contact area between the film and the water droplet. Furthermore, the incorporation of fluoride ions may alter the surface energy by introducing polar sites or modifying the surface chemistry, thereby further promoting wettability. At higher doping concentrations, a slight increase in contact angle is observed, which correlates with the formation of a denser and more compact surface structure. As supported by SEM and AFM data, excessive doping reduces surface roughness and porosity, which limits water spreading and leads to a slight decrease in the hydrophilic character of the films.^49^ These results confirm that NaF doping significantly influences the wetting behavior of Sb_2_S_3_ thin films, with the most favorable wettability achieved at an intermediate doping level.

**Figure 5:**
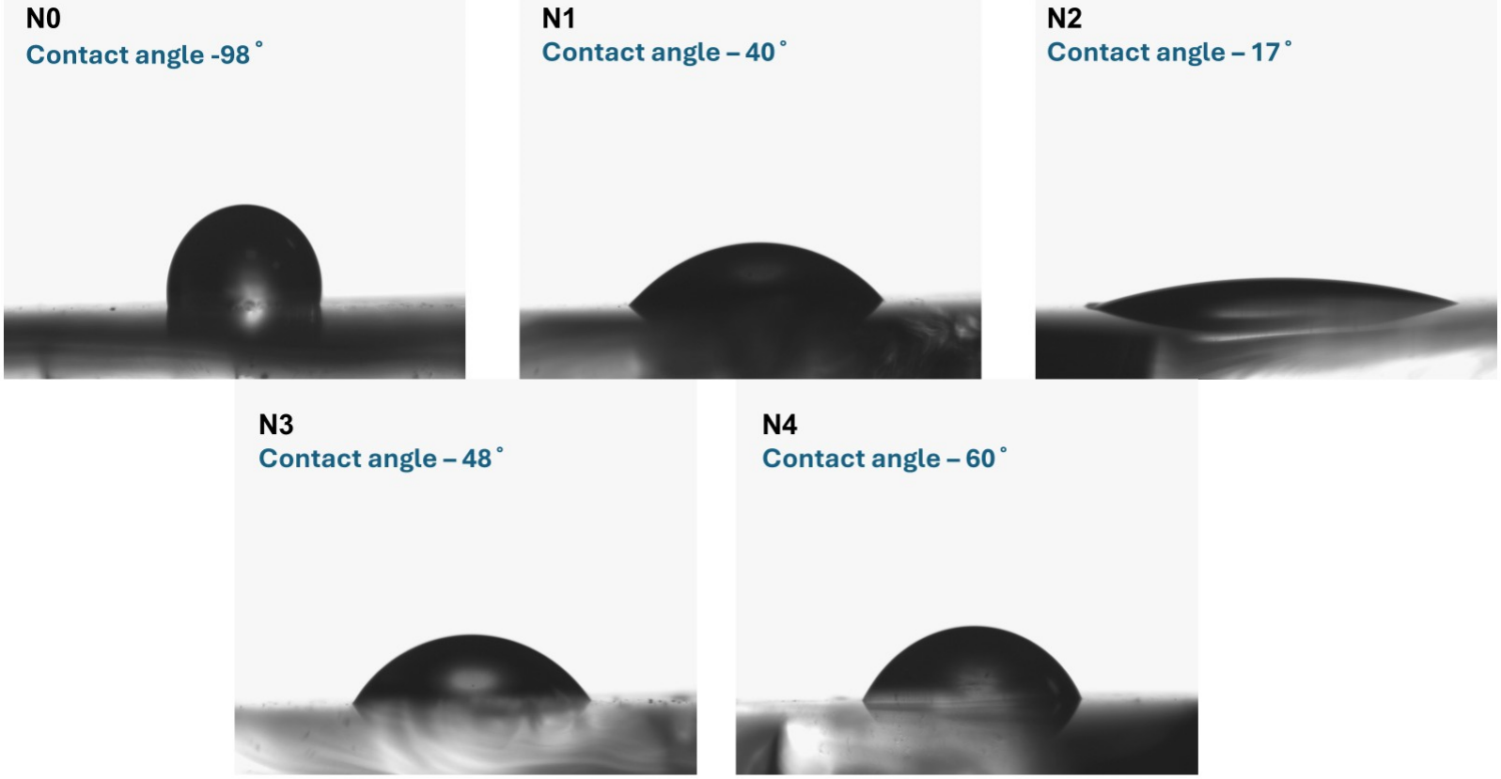
Contact angle of undoped and sodium fluoride-doped antimony sulfide thin films.

### 4.4 Compositional Analysis

Energy-dispersive X-ray spectroscopy (EDS) was used to analyze the elemental composition of sodium fluoride-doped antimony sulfide thin films. Table 4 displays the atomic percentage of each element in the films. The data show that as NaF doping concentration increases, the atomic percentages of sulfur and antimony gradually decrease. This reduction might be due to sodium and fluoride ions partially integrating into or replacing parts of the Sb_2_S_3_ lattice, possibly occupying or distorting existing Sb and S sites. The apparent decrease in sulfur content could also result from volatilization during thermal processing or disruptions in stoichiometry caused by doping. Additionally, accurately measuring fluoride content was not feasible, likely because its low atomic number and interference from the substrate hinder EDS detection of fluorine.

**Table 4:** Atomic Percentage of Undoped and Sodium Fluoride-Doped Antimony Sulfide Thin Films

	atomic percentage (%)		
sample name	Sb	S	Na
N0	33	67	0
N1	30	55	15
N2	29	50	21
N3	26	48	26
N4	26	45	29

Figure 6(a) presents the X-ray photoelectron spectroscopy (XPS) survey spectra of undoped and sodium fluoride-doped antimony sulfide (Sb2S3) thin films, offering detailed information about their surface chemical composition. The binding energy peaks were deconvoluted using a Gaussian–Lorentzian fitting function, and all spectra were calibrated concerning the C 1s peak positioned at 284.6 eV. The survey spectra confirm the presence of Sb, S, Na, and F in the samples. In addition, signals corresponding to C and O are attributed to adventitious carbon and surface-adsorbed oxygen, respectively.

**Figure 6:**
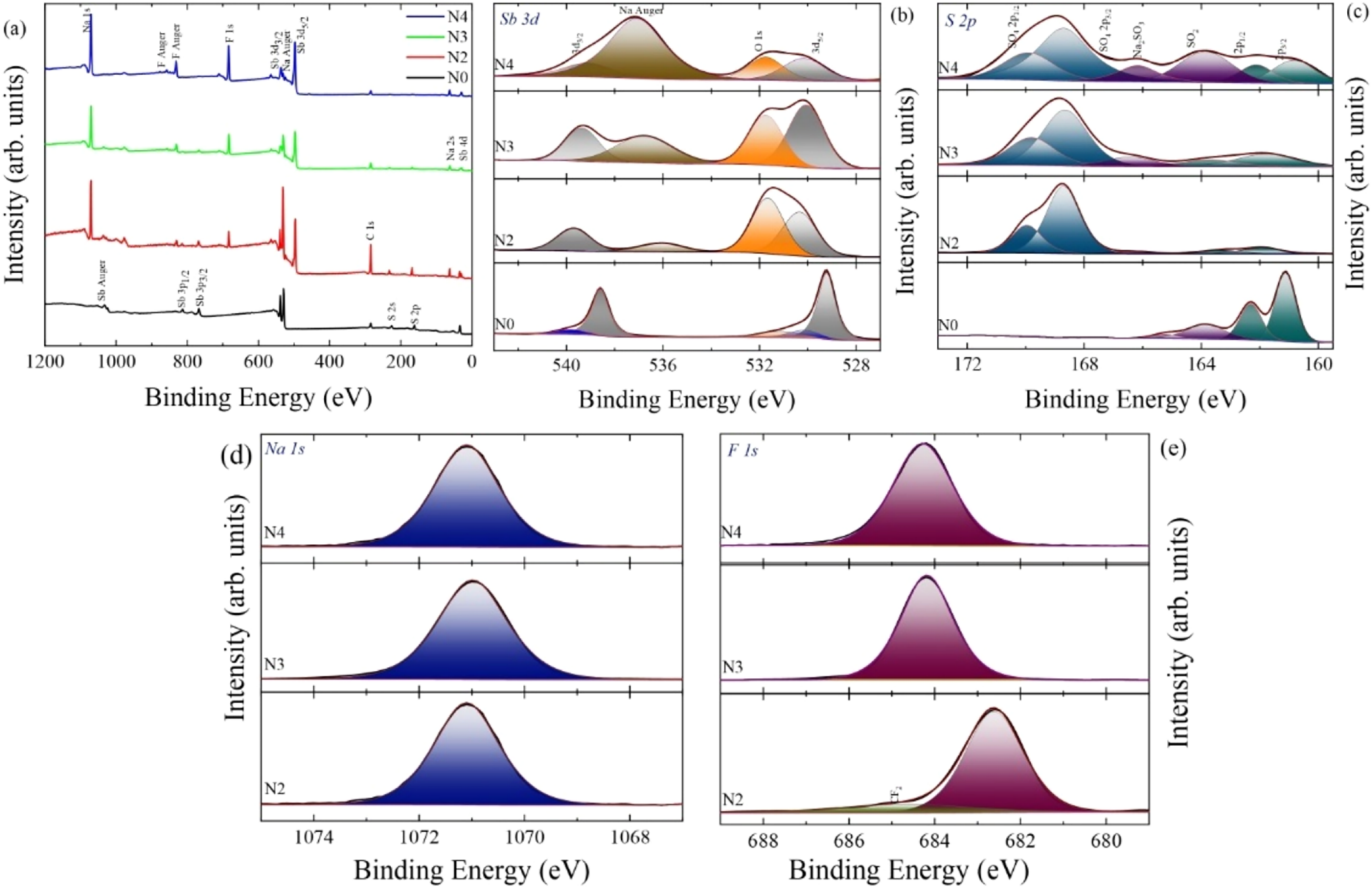
(a) XPS survey spectra, (b–e) core spectra of Sb 3d, S 2p, Na 1s, and F 1s undoped and sodium fluoride-doped antimony sulfide film.

The high-resolution Sb 3d spectrum displays two prominent peaks at binding energies of 539.0 and 529.6 eV, corresponding to the Sb 3d_3/2_ and Sb 3d_5/2_ components of Sb_2_S_3_. The observed spin–orbit splitting of 9.4 eV and the area ratio of approximately 3:2 are consistent with the expected values for antimony in the Sb^3+^ state.^50–52^ A distinct peak at 537.0 eV is assigned to sodium species, confirming successful Na incorporation as a result of NaF doping.^53^ The intensity of this peak increases with higher doping concentrations, indicating a progressive incorporation of sodium into the Sb_2_S_3_ matrix.

Additionally, weak peaks at binding energies of 540.0 and 530.4 eV are attributed to Sb_2_O_3_, suggesting partial surface oxidation.^54^ These oxide-related peaks are present in the undoped sample (N0), indicating that the pristine Sb_2_S_3_ surface is susceptible to oxidation. However, with increasing NaF doping, the intensity of these peaks decreases gradually, reflecting a suppression of surface oxidation. This behavior may be explained by the preferential reaction of available oxygen with sodium and sulfur, rather than with antimony, resulting in the formation of sodium-based sulfur–oxygen compounds.

The S 2p core-level spectrum exhibits well-resolved doublets at 160.8 and 161.9 eV, corresponding to S 2p_3/2_ and S 2p_1/2_ peaks, respectively. The energy separation of 1.1 eV and an area ratio of 2:1 confirm the presence of sulfur in the S^2–^ oxidation state, consistent with the sulfide phase of Sb_2_S_3_.^55,56^ In addition to these primary peaks, weaker features are observed at 163.0 and 164.0 eV, which are attributed to oxidized sulfur species such as sulfur oxides and sulfates.^57^ Peaks at 166.6, 168.5, and 169.8 eV correspond to Na_2_SO_3_, and Na_2_SO_4_, respectively.^58^ These peaks are not present in the undoped sample but appear after NaF doping, suggesting their formation during the postdeposition sulfurization process.

The appearance of these oxidized sulfur species in the doped films is likely due to the interaction between residual oxygen present in the sulfurization chamber and the doped elements (Na and S). This results in the formation of stable sodium–sulfur–oxygen phases. These findings indicate that NaF doping not only alters the chemical composition but also improves the chemical stability of Sb_2_S_3_ films by minimizing antimony oxidation.

Furthermore, the Na 1s core-level spectrum exhibits a clear singlet at a binding energy of 1071 eV, confirming the presence of sodium.^59^ The F 1s core-level spectrum shows a peak at 683 eV, which corresponds to fluorine introduced through NaF.^53^ A shift of approximately 2 eV in the F 1s peak position is observed after the N2 sample. This shift may be attributed to changes in the local chemical environment of fluorine, possibly due to stronger interactions with the Sb_2_S_3_ matrix or changes in the bonding configuration at higher doping levels.

These results collectively confirm that NaF doping influences not only the chemical composition and bonding environment but also modifies the oxidation behavior of both sulfur and antimony species in Sb_2_S_3_ thin films.

### 4.5 Optical Analysis

Figure 7 shows the photoluminescence (PL) spectra of undoped and sodium fluoride-doped antimony sulfide (Sb_2_S_3_) thin films, revealing emission peaks centered at approximately 410, 440, 498, 550, and 630 nm. The peaks in the range of 410 to 498 nm correspond to blue emission, which is commonly attributed to defect-related states and shallow trap levels within the bandgap.^60,61^ The emission observed at 550 nm is assigned to green emission, which is likely associated with sulfur vacancies or surface-related defects.^62^ The broad peak at 630 nm indicates red emission, which may arise from deeper-level defect states.^63^

**Figure 7:**
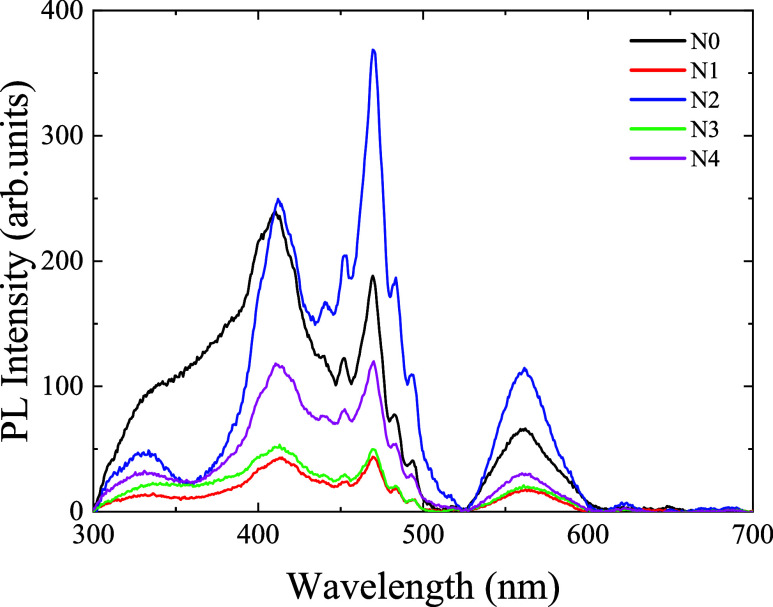
PL Spectra of undoped and sodium fluoride-doped antimony sulfide thin films.

The variation in PL intensity with increasing sodium fluoride doping concentration reflects changes in the recombination behavior of charge carriers. A decrease in PL intensity indicates reduced radiative recombination, suggesting that charge carriers are more frequently undergoing nonradiative recombination pathways. This can be attributed to the introduction of defect states that act as nonradiative recombination centers.^64^

These observations are consistent with the understanding that moderate doping can passivate surface defects and improve crystalline quality, while excessive doping introduces deep-level defects or complex defect clusters that enhance nonradiative losses. Therefore, the quenching of PL emission at higher silver concentrations is likely due to the formation of such recombination centers.

### 4.6 Electrochemical Analysis

Figure 8(a) presents the Nyquist plots obtained from electrochemical impedance spectroscopy (EIS) measurements performed under dark conditions. The semicircle observed in the high-frequency region of each plot corresponds to the charge transfer resistance (*R*_ct_) at the semiconductor-electrolyte interface, as well as the overall resistance of the electrode system. A larger semicircle radius indicates higher charge transfer resistance, while a smaller radius corresponds to improved charge transport between the electrode and the electrolyte.^65^

**Figure 8:**
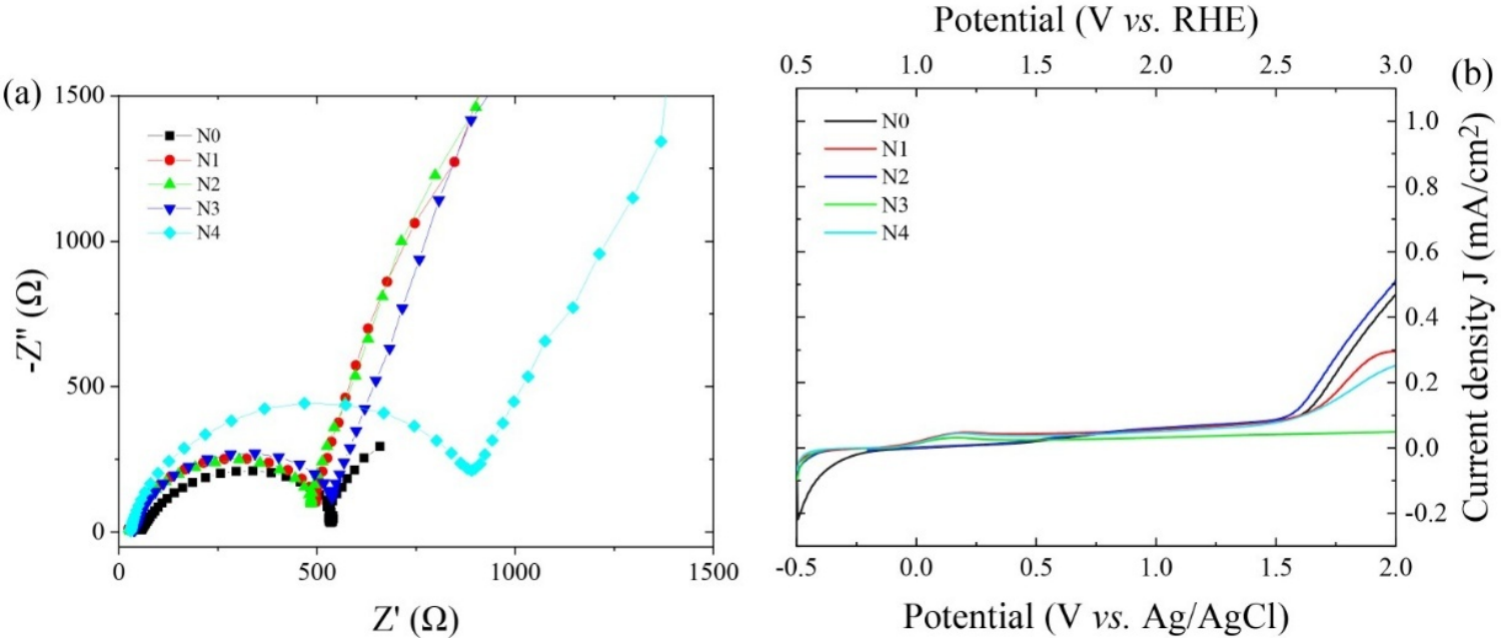
(a) Nyquist plot and (b) LSV curves of undoped and sodium fluoride-doped antimony sulfide thin films.

The undoped Sb_2_S_3_ sample (N0) exhibits a relatively small semicircle, suggesting inherently low charge transfer resistance. Upon doing NaF, a further reduction in the semicircle radius is observed, reaching a minimum for the N2 sample. This indicates that the N2 composition exhibits the most efficient interfacial charge transfer among all samples.

The enhanced performance of the N2 sample can be attributed to several factors. First, the presence of a higher concentration of the Na_2_S phase, as inferred from Raman analysis, likely contributes to improved electrical conductivity. Second, SEM and AFM results confirm that N2 has the highest surface porosity and roughness, respectively, which collectively increase the effective surface area and facilitate better contact with the electrolyte. Additionally, the lowest contact angle observed for this sample indicates superior wettability, promoting efficient charge transfer at the interface.^66^

Beyond the N2 composition, an increase in the semicircle radius is observed with further doping, indicating a decline in charge transfer efficiency. This trend may be explained by the formation of a denser and less porous surface structure, as confirmed by SEM and AFM analyses. Reduced surface roughness and higher contact angles in these samples limit electrolyte accessibility and electron transport across the interface. Furthermore, the S 2p core-level spectra revealed the formation of oxidized sodium sulfur species (Na_2_SO*_X_*) at higher doping levels, which may introduce insulating phases on the surface and hinder charge transfer processes.

Figure 8(b) shows the linear sweep voltammetry (LSV) curves of the undoped and sodium fluoride-doped antimony sulfide (Sb2S3) thin films under illumination, illustrating the influence of NaF doping on photocurrent response. The pristine sample (N0) exhibits a photocurrent density of ∼0.48 ± 0.01 mA cm^–2^. As the NaF doping concentration increases, the photocurrent density also increases, reaching a maximum of about 0.52 ± 0.01 mA cm^–2^ for the N2 sample. The observed improvement can be explained by the combined effects of the structural features and the highly porous surface morphology. AFM measurements confirmed that this composition possesses increased surface roughness. These characteristics collectively result in a larger electrochemically active surface area, which facilitates higher light absorption and enhances charge separation and transport.^67^ In addition, the electrochemical impedance spectroscopy (EIS) results shown in Figure 8(a) reveal that the N2 sample exhibits the lowest charge transfer resistance among all the tested compositions. This indicates more efficient carrier movement across the semiconductor–electrolyte interface, further contributing to the improved photocurrent response.^68^

Beyond the optimal doping level (N2), a gradual decline in photocurrent density is observed. This decrease in performance is consistent with the increase in charge transfer resistance and the reduction in surface roughness and porosity observed in the SEM and AFM analyses. The formation of a more compact surface structure at higher doping levels likely limits charge mobility and reduces interfacial contact with the electrolyte.

Figure 9 presents the Mott–Schottky (M–S) plots, showing the relationship between 1/C2 and applied potential (vs Ag/AgCl) for undoped and sodium fluoride-doped antimony sulfide (Sb2S3) thin films under illumination. These plots are used to estimate the flat-band potential (*V*_fb_) and carrier concentration, which are critical parameters influencing the photoelectrochemical (PEC) performance of semiconductor electrodes in water splitting applications.^69^

**Figure 9:**
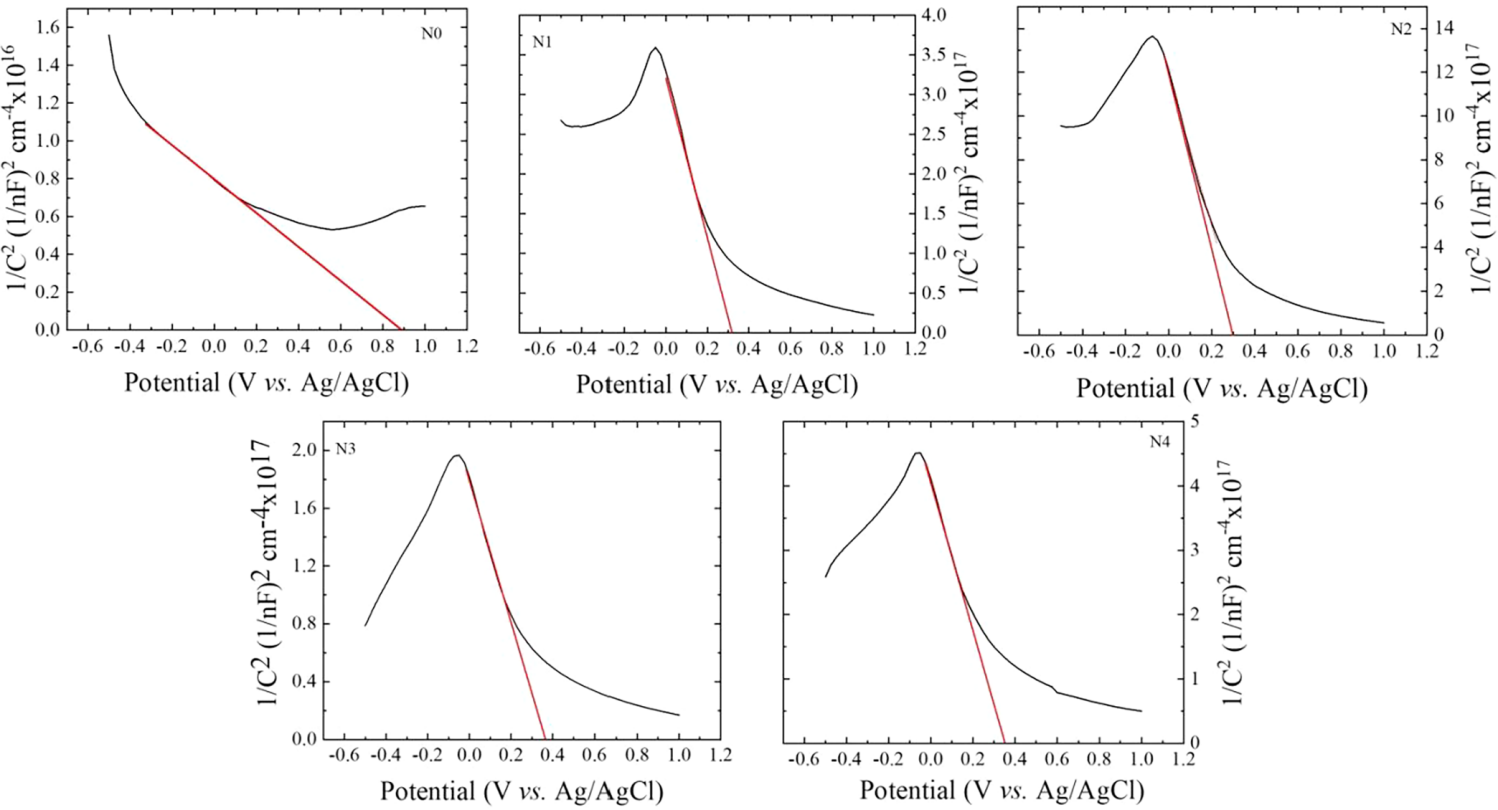
Mott–Schottky curves of sodium fluoride-doped antimony sulfide thin films.

For an ideal n-type semiconductor with uniform donor distribution, the M–S plot typically exhibits a linear region with a positive slope. In the present study, all samples display positive slopes, confirming n-type conductivity. However, minor deviations from linearity are observed, especially in samples with higher doping levels. This nonlinear behavior can be attributed to surface roughness, nonplanar semiconductor–electrolyte interfaces, and inhomogeneous doping, which can lead to irregular charge transport and local potential barriers. In addition, possible ionic adsorption at the surface may alter the interfacial charge distribution, leading to further deviation from ideal linear behavior. These interpretations are supported by previous morphological analyses, which confirmed increased roughness and porosity through AFM and SEM studies.^70^

The flat-band potential for each sample was estimated by extrapolating the linear portion of the M-S plot to the *x*-axis. A slight variation in *V*_fb_ is observed with increasing NaF doping concentration, as shown in Table 5. The variation in *V*_fb_ can be explained as follows. For the pristine sample N0, *V*_fb_ is 0.41 V. As doping begins, *V*_fb_ decreases and reaches 0.33 V for the optimally doped sample. Beyond this level, *V*_fb_ slightly increases to 0.35 V. This change may be related to the decrease in surface roughness, as seen in the AFM images, and the increase in contact angle observed in the contact angle measurements. The shift of *V*_fb_ toward more negative values indicates improved band alignment with the redox potential of water splitting. This alignment reduces the energy barrier for charge transfer between the photoelectrode and the electrolyte, allowing faster interfacial charge exchange and better photoelectrochemical activity. These variations in flat-band potential can be related to dopant-induced changes in surface states and band bending at the semiconductor–electrolyte interface.^71^

**Table 5:** Photochemical Performance of Sodium Fluoride-Doped Antimony Sulfide Thin Films

sample name	flatband potential *V*_fb_ vs Ag/AgCl ± 0.01 V	carrier concentrations (cm^–3^)
N0	0.41	9.24 × 10^15^ ± 0.5 × 10^15^
N1	0.33	1.04 × 10^18^ ± 0.5 × 10^18^
N2	0.33	3.47 × 10^18^ ± 0.5 × 10^18^
N3	0.34	5.05 × 10^17^ ± 0.5 × 10^17^
N4	0.35	1.98 × 10^17^ ± 0.5 × 10^17^

In addition, the carrier (donor) density was calculated from the slopes of the M–S plots. The results indicate a gradual increase in donor density with increasing doping concentration, reaching a maximum in sample N2. This enhancement is consistent with the improved photocurrent density and lower charge transfer resistance observed in the N2 sample (Figure 9a,9b). The increased carrier concentration strengthens the internal electric field within the space-charge region, thereby promoting more efficient separation and transport of photogenerated charge carriers.^72^ Beyond the optimal doping level (N2), a decline in donor density is noted, which may result from defect-induced compensation or saturation of effective dopant incorporation.^73^

## 5 Conclusion

In summary, sodium fluoride-doped antimony sulfide thin films were prepared on FTO substrates using the thermal evaporation method. Grazing incidence X-ray diffraction (GIXRD) confirmed the presence of Na_2_S and SbF_3_ phases, verifying the successful incorporation of NaF into the films. Scanning electron microscopy (SEM) revealed clear changes in surface morphology after doping, with sample N2 showing a more porous and uniform structure compared to the other samples. Atomic force microscopy (AFM) showed that surface roughness increased from 14.1 nm for the pristine film to 20.8 nm for the doped films. Wettability studies indicated improved hydrophilicity after doping, with sample N2 showing the lowest contact angle of 17.47°. Elemental analysis through EDS and XPS confirmed the presence of Na and F, while XPS also revealed Na_2_SO*_X_*-related phases in the doped films. Photoluminescence (PL) results showed an increase in emission intensity after doping, indicating changes in defect states and carrier recombination. Electrochemical impedance spectroscopy (EIS) showed a reduction in charge transfer resistance, with sample N2 having the lowest value among all samples. Photoelectrochemical measurements demonstrated an increase in photocurrent density from 0.49 mA cm^–2^ for the pristine film to 0.52 mA cm^–2^ for the doped film. Mott–Schottky analysis showed a decrease in flat-band potential and an increase in carrier concentration after doping. Sample N2 exhibited a flat-band potential of 0.33 V vs Ag/AgCl and a carrier concentration of 3.47 × 10^18^ cm^–3^. These results show that NaF doping improves the structural, morphological, optical, and photoelectrochemical properties of antimony sulfide thin films. The improved band alignment, higher carrier density, and reduced charge transfer resistance in the doped samples suggest that these films are suitable for use in photoelectrochemical water-splitting devices. The uniform and stable surface morphology, along with the simple and scalable thermal evaporation process, also indicates the potential of these films for large-area device integration and long-term operational stability.
